# Oropharyngeal Hairy Polyp: A Case of Respiratory Failure in a Newborn

**DOI:** 10.3390/diagnostics10070465

**Published:** 2020-07-09

**Authors:** Paola Feraco, Emma Bragantini, Francesca Incandela, Cesare Gagliardo, Marina Silvestrini

**Affiliations:** 1 Neuroradiology Unit, S. Chiara Hospital, Largo Medaglie d’oro 9, 38122 Trento, Italy; 2 Department of Surgical Pathology, S. Chiara Hospital, Largo Medaglie d’oro 9, 38122 Trento, Italy; emma.bragantini@apss.tn.it; 3 Section of Radiological Sciences, Department of Biomedicine, Neuroscience and Advanced Diagnostics, University of Palermo, Via Del Vespro, 129, 90127 Palermo, Italy; francescainch@gmail.com (F.I.); cesare.gagliardo@unipa.it (C.G.); 4 Department of Otorhinolaryngology, Head and Neck Surgery, San Bortolo Hospital, Viale Ridolfi 37, 36100 Vicenza, Italy; marina.silvestrini@gmail.com

**Keywords:** oropharyngeal dermoid, respiratory distress, airway obstruction, magnetic resonance imaging (MRI)

## Abstract

Hairy polyps, also known as dermoid polyps (DPs), are rare benign cystic lesions of bigerminal origin that may occur in several head and neck regions, including the oropharynx. Despite their benign histopathological nature, DPs may be life threatening, due to their upper airway location, and DPs represent one of the most unusual causes of respiratory distress during the neonatal period. In this paper, we describe a case of respiratory failure in a newborn with an oropharyngeal mass that was accidentally found during difficult intubation. Magnetic resonance imaging (MRI) detected a well-defined soft tissue pedunculated mass, arising from the left oropharynx wall, consistent with an oropharyngeal DP. The newborn had a prompt recovery after trans-oral mass removal. Our case underlines the importance of imaging in differential diagnosis of children’s respiratory distress, secondary to a variety of lesions within the region of the skull base or oropharynx. It allowed us to assess the origin of the lesion, as well as its relationship with the adjacent soft tissues, and to exclude intracranial extension, thus providing essential information for the surgical planning.

**Figure 1:**
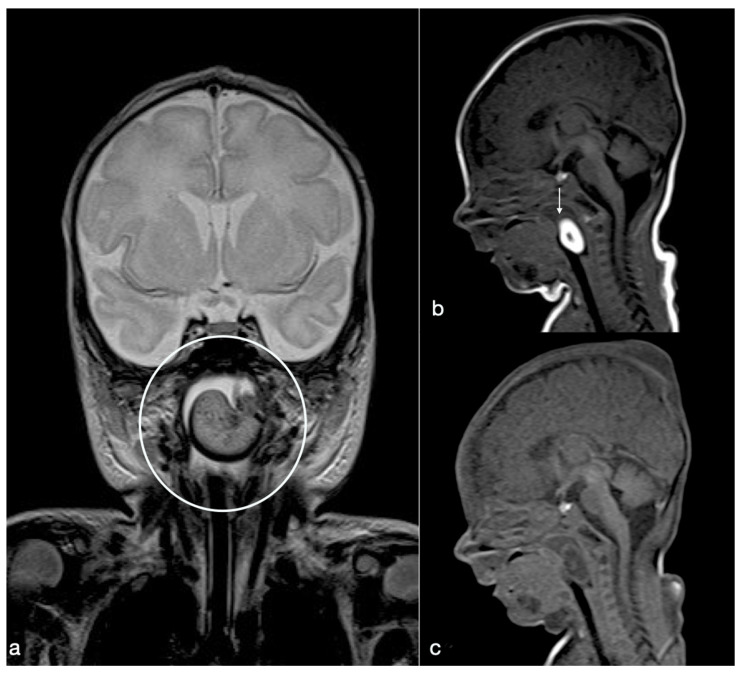
We report the case of respiratory failure in a male newborn, whose pregnancy was unknown until the third trimester when it was discovered due to polyhydramnios. At 38th gestational week, caesarean section was needed due to fetal suffering: he was born pale, floppy, hypotonic, not reagent to external stimuli, with a low heart rate (HR (heart rate) < 60 bpm), not responsive to medical treatment, and showing increasing oxygen desaturation up to respiratory distress. The intubation was challenging, so physicians performed a fiber-optically controlled one, demonstrating a well-defined obstructive nasal-oropharyngeal soft white mass. After vital parameters stabilization, he was immediately transferred to the neonatal intensive care. On physical examination, no abnormalities were noticed; also, cardiac, pulmonary, and abdominal examinations were normal. He promptly underwent magnetic resonance imaging (MRI) investigation to define the lesion. MRI of head and neck revealed a well-defined soft tissue pedunculated mass (2.5 × 2 × 1 cm), arising from the left nasal-oropharynx wall, occupying the space between the epiglottis and the uvula in the midline. In the T2-weighted coronal section (**a**), it appears hyperintense with small punctate hypointense foci in the center and hypointense peripherally (open circle). A T1-weighted image obtained on the sagittal plane (**b**) shows a hyperintense “pearly” appearance of the lesion (arrow), with the exception of a small hypointense center. In the unenhanced, fat-saturated sagittal T1-weighted image (**c**), the mass is completely hypointense, consistent with a fat-rich content. These findings were consistent with oropharyngeal dermoid polyp (DP); in particular, a DP does not show restricted diffusion, contrary to an epidermoid cyst [1]. DPs have to be differentiated by other lesions within the region of the skull base or pharynx, which may cause children’s respiratory distress, such as meningoencephalocele, neuroglial heterotopias, and gliomas [1,2,3]. In particular, a DP differs from a meningoencephalocele, which is hypointense on T1-weighted and FLAIR images (similar to brain tissue), showing a direct communication through a skull base defect with the cranial vault; or from neuroglial heterotopy, which shows T1 and T2 signal characteristics of brain gray and white matter with small enclosed cysts; instead of gliomas, which usually show a hypointense signal on T1-weighted images [1]. Even if the use of ultrasound is useful for the first-line investigation of neck masses in neonates [4], considering the severity of the respiratory failure, our patient directly underwent MRI examination for optimal lesion characterization and surgical planning. Moreover, MRI allowed us to assess the relationship of the lesion with the adjacent soft tissues, to depict adjacent bony changes [3,5], and to exclude intracranial extension, thus providing essential information for the surgical planning [2]. This approach is essential in terms of surgical management and outcome. In our case, there were neither underlying bony skull base defects, nor intracranial extension. No cervical enlarged lymph nodes were found.

**Figure 2:**
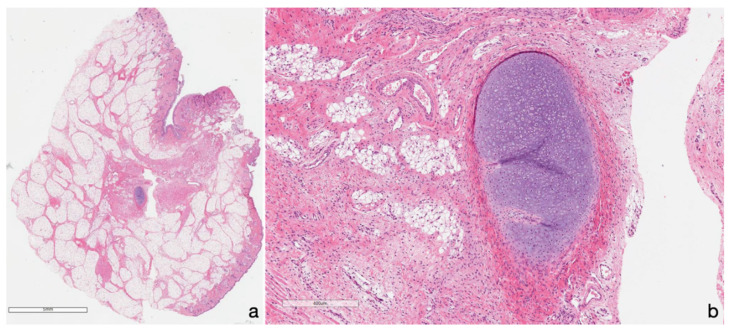
The newborn underwent surgical trans-oral mass removal, with consequent prompt recovery during hospitalization. Non-residual mass was detected during the radiological follow-up. The histologic assessment confirmed MRI suspicion, revealing a stratified keratinized epithelium mass, containing dermal skin appendages as sebaceous and sudoriferous glands, mixed to adipose tissue (**a**) with a small cartilaginous fragment (as shown in detail in **b**), consistent with hairy polyp (also known as dermoid polyps (DPs)). It is a benign epithelial inclusion cystic lesion [1,2,3,4,5,6,7] resulting from abnormal separation of ectoderm and neuroectoderm along the lines of embryonic fusion, during the first steps of embryogenesis, between the third–fifth gestational week [6,7]. Dermoids may occur in the head and neck region, in particular, settled in the orbital region, nasal dorsum, mouth floor, infratemporal fossa, nasopharynx, oropharynx, and anterior and lateral part of the neck, also arising from the eustachian tube [3], although they may be found anywhere on the body [5]. DPs are frequently found in the neonatal period, but occasionally they can be seen in older children, since a DP’s clinical presentation depends on the site of origin and on its size [2]. When in the naso-oropharyngeal cavity, as in our case, DPs can represent a rare cause of upper airway obstruction, ranging from intermittent symptoms or even respiratory distress [1,2]. Other clinical presentations described are feeding disorders with vomits, hemoptysis, hearing loss, otorrhagia, snoring, and recurrent ear infections [1,8]. Pregnancy could be normal, but polyhydramnios can still occur because of the obstruction of the swallowing mechanism, as in our case [2,9]. Although most of these lesions are superficial, variable rates of intracranial extension have been reported [5,10], above all, in those of the midline neck, which have also been associated with cranial or spinal dysraphism [5,11]. The case discussed above reported an oropharyngeal DP, occupying the space between the epiglottis and the uvula; despite its midline setting, it had neither intracranial extension nor dysraphisms. In addition, DPs are rarely associated with other congenital abnormalities, such as facial hemihypotrophy, ankyloglossia, cleft palate, absent uvula, auricle deformities, or left carotid artery atresia [2], so congenital malformations should be investigated. In our case, the physical examination was normal, and further investigations detected all associated anomalies. The standard of DP management when symptomatic is excision without disruption of the cyst wall. Indeed, if the cyst wall is ruptured at the time of surgery, the remnant tissue should be removed using curettage and copious irrigation [5]. DP prognosis has been shown to be excellent, without recurrence after excision [2,6], as reported in our case. This case report adds another case of this rare pathology to the literature. DPs should be kept in mind in case of newborn respiratory failure. Despite their benign histopathological nature, DPs may be life threatening, since they represent one of the most unusual causes of airway obstruction.
